# Role of Peroxisome Proliferator-Activated Receptor Gamma and Its Ligands in the Treatment of Hematological Malignancies

**DOI:** 10.1155/2008/834612

**Published:** 2008-05-26

**Authors:** Tatiana M. Garcia-Bates, Geniece M. Lehmann, Patricia J. Simpson-Haidaris, Steven H. Bernstein, Patricia J. Sime, Richard P. Phipps

**Affiliations:** ^1^Department of Microbiology and Immunology, University of Rochester School of Medicine and Dentistry, Rochester, NY 14642, USA; ^2^Department of Environmental Medicine, University of Rochester School of Medicine and Dentistry, Rochester, NY 14642, USA; ^3^Department of Medicine, University of Rochester School of Medicine and Dentistry, Rochester, NY 14642, USA; ^4^Department of Pathology and Laboratory Medicine, University of Rochester School of Medicine and Dentistry, Rochester, NY 14642, USA; ^5^The Lymphoma Biology Program of James P. Wilmot Cancer Center, University of Rochester School of Medicine and Dentistry, Rochester, NY 14642, USA; ^6^The Lung Biology and Disease Program, University of Rochester School of Medicine and Dentistry, Rochester, NY 14642, USA

## Abstract

Peroxisome proliferator-activated receptor gamma (PPAR*γ*) is a multifunctional transcription factor with important regulatory roles in inflammation, cellular growth, differentiation, and apoptosis. PPAR*γ* is expressed in a variety of immune cells as well as in numerous leukemias and lymphomas. Here, we review recent studies that provide new insights into the mechanisms by which PPAR*γ* ligands influence hematological malignant cell growth, differentiation, and survival. Understanding the diverse properties of PPAR*γ* ligands is crucial for the development of new therapeutic approaches for hematological malignancies.

## 1. INTRODUCTION

In order to understand the influence of PPAR*γ* and its many ligands on hematological malignancies and their normal cell counterparts, we first present background material to orient the reader.

Peroxisome proliferator-activated receptors (PPARs) *α*, *β*/*δ*, and *γ* are members of the nuclear hormone receptor superfamily of transcription factors that regulate several metabolic pathways in a tissue-selective manner [1]. All PPARs form heterodimers with members of the retinoid X receptor (RXR) subfamily of nuclear hormone receptors and regulate initiation of transcription by binding to the peroxisome proliferator response element (PPRE) in promoters of target genes. Drug classes such as fibrates and thiazolidinediones are used for lowering lipids and improving insulin sensitivity, respectively, thus effectively reducing risk factors that lead to cardiovascular disease [2, 3] and diabetes [4, 5]. PPAR*γ* agonists have both PPAR*γ*-dependent and -independent effects on coagulation, thrombosis, angiogenesis, and tumor growth and metastasis [6, 7]. PPAR*γ* agonists also exert anti-inflammatory and antifibrotic effects by negatively regulating the expression of proinflammatory genes and by inhibiting myofibroblast differentiation [8–10]. Moreover, PPAR*γ* agonists modulate the activity of several transcription factors (e.g., NF-*κ*B, AP-1, and Stat3) [10–13] that regulate inflammation.

### 1.1. Structure of the human PPAR*γ* gene

**Figure 1:**
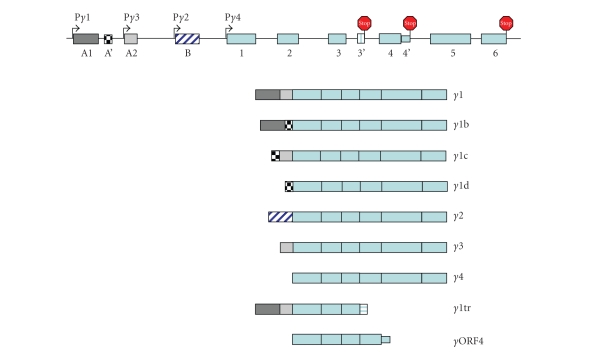
*Schematic structure of the human PPAR*γ* gene.* The human PPAR*γ* gene is located on chromosome 3, band 3p25, and is composed of at least 11 exons that give rise to 9 transcript variants. Expression of PPAR*γ* involves differential promoter usage in combination with alternative splicing and polyadenylation site selection. The relative positions of the four known PPAR*γ* promoters are designated as P*γ*1-P*γ*4. The noncoding exons A1, A′, and A2 are depicted by boxes in different shades of gray or in black and white checked. These exons contribute to the 5′ UTR of transcripts *γ*1-*γ*1d, *γ*3 and *γ*1tr. The transcript variants 1*γ*-1*γ*d, *γ*3, and *γ*4 encode the PPAR*γ*1 isoform. Exon B (diagonal blue and white hatched box) encodes the 28 additional amino acids found at the amino terminus of human PPAR*γ*2; the mouse PPAR*γ*2 exon B encodes 30 amino acids. Exons 1–6 (light blue boxes) are common in all PPAR*γ*1 transcripts and when they are spliced to exon B encode full-length PPAR*γ*2. Two additional exon regions have been recently identified, exon 3′ (horizontal light blue and white hatched box) and exon 4′ (small light blue box). Inclusion of either of these coding regions in the processed mRNA transcript results in truncated PPAR*γ*1 proteins lacking the ligand binding domain (*γ*1tr and ORF4, resp.). The sizes of the exon boxes approximate the relative lengths of each exon; however, the introns (depicted as straight lines) are not drawn to scale. The positions of the stop codons are depicted by the hexagonal red stop signs.

The human PPAR*γ* is located on chromosome 3, band 3p25 [14]. This gene gives rise to the two well-known isoforms of PPAR*γ*, PPAR*γ*1, and PPAR*γ*2, which function as transcriptional activators or repressors in a context-dependent manner [15, 16]. Recent evidence suggests that the human PPAR*γ* gene is composed of at least 11 exons that give rise to 9 transcript variants due to the combination of differential promoter usage, alternative RNA splicing, and polyadenylation site selection of the primary transcript (Figure 1). To date, four promoters and three new exons A′, 3′, and 4′ have been identified [14, 17–23]. Similar to exons A1 and A2, exon A′ is noncoding and contributes to the 5′ UTR of several transcript variants (Figure 1). Inclusion of exon 3′ in the processed transcript produces a truncated PPAR*γ*1 protein (*γ*1tr) [22], as does the read-through of exon 4 to include intron 4 sequences (*γ*ORF4) [23]. Both truncated forms of PPAR*γ*1 (*γ*1tr and *γ*ORF4) lack the coding regions for the ligand binding domain and function in a dominant negative manner to wild type PPAR*γ*1. The truncated form of PPAR*γ* (*γ*1tr) was discovered and cloned from chronic myeloid leukemia K562 cells and enhanced cell proliferation [22]. Similarly, *γ*ORF4 protein was found to reside mainly in the nucleus and enhanced cell growth [23]. The complexity in processing the PPAR*γ* primary transcript likely leads to specific regulation of PPAR*γ* functions in a context-dependent manner. This may explain, at least in part, the pleiotropic functions ascribed to PPAR*γ*1 and PPAR*γ*2 [23–29].

### 1.2. Posttranslational modifications regulate PPAR*γ* activity

**Figure 2:**
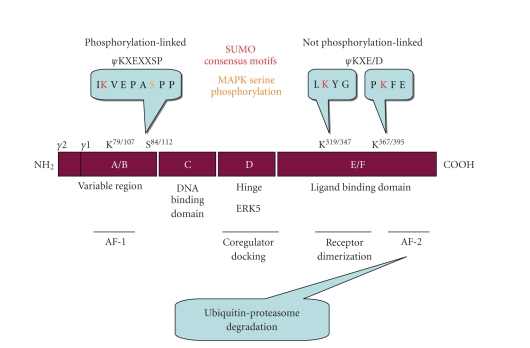
*Reversible posttranslational modifications of PPAR*γ**. The superfamily of nuclear hormone receptors possesses conserved structural and functional domains including PPAR*γ*. The A/B domain is the hypervariable region containing the putative activation function-1 (AF-1) domain. Human PPAR*γ*2 contains a 28 amino acid amino terminal region that arises from differential promoter use and splicing (see Figure 1). The primary structure of the C-domain is the most conserved and contains the DNA binding domain (DBD). The D-domain (Hinge) allows for conformational change following ligand binding to promote coregulator (coactivator or corepressor) docking; binding of ERK5 to the hinge helix 1 region potentiates ligand-dependent PPAR*γ*1 activity. The E/F region contains the ligand binding domain (LBD) of PPAR*γ* and the activation function-2 (AF-2) domain that participates in ligand-dependent degradation mediated by the ubiquitin-proteasome pathway. PPAR*γ* heterodimerizes with its binding partners, RXR family members, through the E/F domain as well. Reversible posttranslational modifications of PPAR*γ* regulate its activation. In addition to proteasome-mediated degradation, PPAR*γ* can be phosphorylated by MAP kinases at S^84/112^ (position of serine in PPAR*γ*1/PPAR*γ*2) or SUMO-1 modification. Two SUMOylation consensus motifs have been described. Whereas SUMOylation at a conserved *ψ*KXEXXSP (where *ψ* is a hydrophobic amino acid and X can be any residue) is linked to serine phosphorylation events, SUMOylation at *ψ*KXE/D motifs are not generally linked to MAPK phosphorylation. The lysine residues of the three SUMOylation motifs identified on PPAR*γ*1/2 are depicted in red. The serine residue phosphorylated by MAPKs is depicted in yellow. Both serine phosphorylation and SUMOylation negatively regulate PPAR*γ* activity.

Several reversible posttranslational modifications occur that regulate the transactivation potential of PPAR*γ* (Figure 2). The phosphorylation status and activity of the PPARs are regulated in both ligand-dependent and ligand-independent manners, the details of which have been recently reviewed [30]. Whereas serine phosphorylation of PPAR*α* increases its transcriptional activity in hepatocytes, MAPK/ERK-mediated phosphorylation of Ser^84/112^ on PPAR*γ*1/2 leads to attenuation of PPAR*γ* transcriptional activity and its possible relocalization from the nucleus to the cytoplasm [30–33]. Furthermore, both Ser^84/112^ phosphorylation [34] and ligand binding [35] contribute to the targeting of PPAR*γ* to ubiquitin-proteasome degradation. In contrast, ERK5 activates PPAR*γ*1 in a phosphorylation-independent manner by directly interacting with the hinge-helix 1 region [36].

In a recent review, Straus and Glass [10] discuss various mechanisms for nuclear hormone receptor-dependent transrepression of target genes by the PPARs, Liver X Receptors (LXRs), and glucocorticoid receptor (GR). Posttranslational modification with small ubiquitin-like modifier (SUMO)-1 converts these nuclear hormones from transactivators to transrepressors of gene expression [10, 37]. SUMOylated PPAR*γ*1 binds to the corepressor complex interfering with its clearance, thereby preventing transactivation of NF-*κ*B target genes [10, 37]. To date, modifications of PPAR*γ* with SUMO-1 occur on three lysine residues (K^79/107^, K^319/347^, and K^367/395^) of PPAR*γ*1/2 [38–40]. Moreover, PPAR*γ*'s dimerization partner, RXR*α*, is also SUMOylated [41]. A summary of PPAR*γ* posttranslational modifications is shown in Figure 2. SUMO competes with ubiquitin for modification of lysines on some proteins, thereby rescuing the protein from ubiquitin-proteasome mediated proteolysis [42]. In addition to increasing protein half-life, SUMOylation plays a role in nuclear-cytoplasmic trafficking, cell-cycle regulation, genome integrity, transcription, and cancer progression and metastasis [43–47].

## 2. PPAR*γ* LIGANDS

Transcriptional activity of PPAR*γ* is controlled primarily by ligand binding [48]. PPAR*γ* has a large ligand binding pocket, which enables it to bind a variety of ligands [49]. PPAR*γ* ligands include both synthetic and natural molecules [48]. Many of the naturally occurring ligands are fatty acids or fatty acid derivatives obtained through the diet or from intracellular signaling pathways. These include lysophosphatidic acid [50], nitrolinoleic acid [51], 9- and 13-hydroxyoctadecadienoic acids (9- and 13-HODE) [48, 52], 15-hydroxyeicosatetraenoic acid (15-HETE) [25], prostaglandin D_2_ (PGD_2_), and 15-deoxy-Δ^12,14^-prostaglandin J_2_ (15d-PGJ_2_) [25, 48, 49, 53–55]. 15d-PGJ_2_ is thought to be the most potent endogenous ligand for PPAR*γ*, activating it at low micromolar concentrations [25, 52, 53]. PGD_2_ and 15d-PGJ_2_ are derived from arachidonic acid by the catalytic activities of the cyclooxygenase-2 (Cox-2) and prostaglandin D synthase [53, 54, 56]. PGD_2_ spontaneously undergoes a series of dehydration reactions to form the PGJ family of prostaglandins, including 15d-PGJ_2_, and 15d-PGD_2_, which can also transactivate PPAR*γ* [56–60].

Synthetic PPAR*γ* ligands, including drugs of the thiazolidinedione (TZD) family (e.g., ciglitazone, pioglitazone, rosiglitazone, and troglitazone), have potent insulin-sensitizing properties [3, 25, 49, 56, 61, 62]. Because of this, some are commonly used for the treatment of type 2 diabetes [48, 61]. There also exist TZDs, such as TZD18, that act as dual PPAR*α*/PPAR*γ* agonists [63].

There are also many non-TZD synthetic compounds that can function as PPAR*γ* agonists. Some of these are: L-tyrosine-based GW-7845 and GW-1929 [48, 52], diindolymethane analogs [48, 64], certain nonsteroidal anti-inflammatory drugs (NSAIDs) (i.e., indomethacin, ibuprofen, flufenamic acid, and fenoprofen [25, 27, 65]), and the novel synthetic triterpenoid 2-cyano-3,12-dioxooleana-1,9-dien-28-oic acid (CDDO) and its derivatives [48, 66]. CDDO binds to PPAR*γ* with nanomolar affinity [48, 66] and displays antiproliferative and differentiating activities, making it useful as a chemotherapeutic agent. Derivatives of CDDO have more useful pharmacodynamic and pharmacokinetic properties than CDDO itself [67, 68]. Importantly, some CDDO derivatives are orally active and are remarkably well-tolerated in humans [69].

PPAR*γ* ligands, including CDDO, can reduce cell proliferation, migration, cytokine production, expression of costimulatory, and adhesion molecules and can promote apoptosis [48]. These findings suggest that PPAR*γ* ligands may be efficacious in the treatment of hematological malignancies [48]. However, numerous side effects have been observed in patients treated with TZDs [49]. For example, troglitazone has caused hepatotoxicity [49]. TZDs also induce weight gain, edema [70], increased lipoprotein(a) concentrations [3, 49], and probably enhance risk of heart failure and cardiac hypertrophy [48, 71, 72]. Therefore, it is highly desirable to develop PPAR*γ* ligands with improved therapeutic profiles [48].

The identification of “selective PPAR*γ* modulators” (SPPAR*γ*Ms) has become the object of intense recent interest, with the idea that one might modulate the genes necessary to achieve therapeutic potential, while not affecting the genes involved in producing side effects [49]. This concept is plausible because SPPAR*γ*Ms take advantage of the large PPAR*γ* ligand binding pocket, which allows a variety of ligands to bind in different orientations [15, 61, 73–76]. SPPAR*γ*Ms then induce specific conformational changes of the receptor which create different interaction surfaces, favoring the recruitment of only a subset of coregulators [48, 49, 77, 78]. This subset of coregulators will allow the induction of some, but not all target genes [15, 49, 61, 74, 79–83]. The SPPAR*γ*M concept has been shown to hold true for some currently recognized PPAR*γ* ligands. For example, CDDO is a more potent inducer of apoptosis than are TZDs [48]. This may be because the PPAR*γ* target genes activated by CDDO are different from those activated by TZDs [48]. CDDO is less effective than rosiglitazone in recruiting coactivators, but it can effectively promote the release of corepressors from PPAR*γ* target genes [48]. A greater understanding of the activities of the various PPAR*γ* ligands will depend on the identification of the specific coregulators recruited to PPAR*γ* target genes in response to binding to specific ligands [25].

## 3. PPAR*γ* AND THE IMMUNE SYSTEM

One of the earliest indications of an important role for PPAR*γ* in the immune system was the discovery of its expression in mouse spleen [84]. After this finding, our laboratory and others began searching for PPAR*γ* expression and function in immune cells. To date, PPAR*γ* expression has been found in monocytes/macrophages, dendritic cells, granulocytes (i.e., neutrophils, eosinophils, and basophils), mast cells, T cells, and B cells, and most recently our laboratory found PPAR*γ* in human platelets [84–90].

PPAR*γ* ligands have been shown to have anti-inflammatory effects on cells of the innate and adaptive immune system [91–94]. In macrophages, PPAR*γ* has an important role in regulating lipid metabolism, as well as in the generation of macrophage-derived foam cells in atherosclerotic lesions [95–98]. Upon phorbol myristyl acetate (PMA) stimulation, PPAR*γ* ligands can inhibit macrophage activation and production of inflammatory cytokines (e.g., TNF*α*, IL-1*β*, and IL-6), inducible nitric oxide synthase (iNOS), gelatinase B, and scavenger receptor A (SR-A) [89, 99, 100]. Moreover, PPAR*γ* activation can skew macrophage differentiation into a more anti-inflammatory phenotype [101]. In dendritic cells, PPAR*γ* activation can inhibit the production of IL-12 and of chemokines involved in the recruitment of Th1 lymphocytes, therefore, favoring a type 2 immune response [90]. PPAR*γ* ligands also enhanced the development of a dendritic cell phenotype that: (1) has increased endocytic activity and (2) induces the expansion of invariant natural killer T (NKT) cells [102].

PPAR*γ* also plays a role in T lymphocyte function, and its levels are upregulated following their activation [103, 104]. PPAR*γ* expression and activation can inhibit T lymphocyte proliferation and reduce the production of IFN*γ*, TNF*α*, and IL-2 [92, 105, 106]. These inhibitory effects result from the direct interaction between PPAR*γ* and the transcription factor nuclear factor of activated T cells (NFAT) [107]. Our laboratory demonstrated that mouse and human T cells express PPAR*γ*, and treatment with PPAR*γ* ligands induces apoptosis in malignant T cells [103, 104]. Recent findings reported by Wohlfert et al. could illuminate yet another regulatory role for PPAR*γ* in the immune system [108]. In their study, PPAR*γ* activation enhanced the generation of CD4^+^ CD25^+^ regulatory T cells (Tregs). Tregs have been demonstrated to play a key role in negatively regulating autoimmunity and immune responses [109]. There are two different subtypes of Tregs: thymus-derived natural Tregs (nTregs) and inducible Tregs (iTregs), which develop from CD4^+^ CD25^−^ effector T cells in the periphery. [109–111]. Wohlfert et al. showed that ciglitazone enhanced the conversion of effector T lymphocytes into inducible Tregs (iTregs). Moreover, PPAR*γ* expression in natural Tregs (nTregs) was required for the in vivo effects of ligand treatment in a murine model of graft versus host disease [108]. These findings suggest that PPAR*γ* ligands enhance the activity of Tregs while dampening the activation of other T lymphocyte subsets. PPAR*γ* was also shown to have a physiological role in regulating B lymphocyte function. In studies using PPAR*γ* haploinsufficient mice, B lymphocytes exhibited increased proliferation and survival, enhanced antigen specific immune responses and spontaneous NF-*κ*B activation [15, 112]. Our laboratory demonstrated that both normal and malignant B lymphocytes express PPAR*γ*, and that exposure to certain PPAR*γ* ligands inhibits B cell proliferation and can induce apoptosis [85, 93, 113].

In summary, PPAR*γ* activation has antiproliferative and proapoptotic effects and dampens cytokine production in several immune cells. PPAR*γ* ligands can also attenuate several inflammatory diseases such as inflammatory bowel disease [114–119], multiple sclerosis [120–122], rheumatoid arthritis [112, 123], and psoriasis [124–126]. These findings suggest that PPAR*γ* ligands may be useful for the treatment of immunological diseases, which include myelo and lymphoproliferative disorders.

## 4. PPAR*γ* AND ITS CONTROVERSIAL ROLE AS A TUMOR SUPPRESSOR GENE

As evidence accumulated to support that PPAR*γ* ligands are inhibitors of cell proliferation and inducers of cell differentiation, attention turned to the role of PPAR*γ* in the onset and development of cancer. The potential of PPAR*γ* ligands as anticancer drug therapies has been explored in cells from various malignant tissues, including those of adipose, colon, breast, prostate, lung, pancreas, bladder, and stomach origin [26, 127]. There is emerging evidence for a direct role of PPAR*γ* functional mutations in the initiation of several common human cancers, such as colon, prostate, and thyroid [28, 128–130]. For example, in a study of 55 patients with sporadic colon cancers, four somatic PPAR*γ* mutations were found. [129]. Also, a hemizygous deletion of PPAR*γ* was identified in 40% of prostate cancers [128]. Furthermore, a fusion protein derived from the paired box gene 8 (PAX8) and PPAR*γ* genes (PPAR*γ*-PAX-8) was detected in thyroid cancers, which causes PPAR*γ* not only to be functionally inactive but also to function as a dominant negative form of PPAR*γ* [28, 131]. As described earlier, the PPAR*γ* gene is mapped to human chromosome 3, band 3p25 [14]. Interestingly, 3p deletions have been identified in several hematological cancers, including acute myeloid leukemias (AML), myelodysplastic syndromes (MDS), Philadelphia chromosome-positive chronic myeloid leukemia (CML), acute lymphoblastic leukemias (ALL), chronic lymphoproliferative disorder (CLD), and non-Hodgkin's lymphomas (NHL) [132]. These observations suggest that PPAR*γ* plays a role as a tumor suppressor gene and, as such, may be a therapeutic target for cancer. Studies in liposarcoma [133] and in xenograft models of prostate [134] and colon cancer [135] support this hypothesis. However, a study using a large number of human tumor samples and cell lines (*n* = 397), including those from leukemias, found no detectable abnormalities, either in PPAR*γ* exon 3 (DBD) or in exon 5 (LBD), suggesting that PPAR*γ* gene mutations may occur, but are rare [136].

The expression levels and/or the transactivation of PPAR*γ* may be impaired in certain cancers. In human lung cancer, decreased expression of PPAR*γ* correlated with poor prognosis [29] and well-differentiated adenocarcinomas had more PPAR*γ* expression than poorly differentiated varieties [137]. In addition, a study performed by Jansen et al. demonstrated that the abnormal PML-RAR*α* (promyelocytic leukemia-retinoic acid receptor alpha) fusion protein found in acute promyelocitic leukemia (APL) interferes with PPAR function [138]. Similarly, Hamadani et al. showed that different X-RAR*α* fusion proteins found in APL can inhibit the transactivation of PPAR*γ*, and that this repression can be released by the addition of PPAR*γ* ligands [139, 140]. These findings suggest that (1) PPAR*γ* may be inactive in APL, (2) this may contribute to the undifferentiated phenotype, and (3) PPAR*γ* ligands may help sensitize APL cells to the differentiating effects of all-*trans*-retinoic acid (ATRA).

## 5. PPAR*γ* AND PPAR*γ* LIGANDS AS POTENTIAL THERAPY FOR HEMATOLOGICAL MALIGNANCIES

### 5.1. Myeloid malignancies

#### 5.1.1. Acute myeloid leukemia (AML)

Acute myelogenous leukemia (AML) constitutes about 25% of all leukemias in adults in the Western World. It ranks as the second most frequent type of leukemia in adults after chronic lymphocytic leukemia, with more than 13000 new cases, and nearly 9000 deaths from AML in the U.S. in 2007 [141]. Unfortunately, this type of leukemia has one of the lowest survival rates, about 20% [142]. There are several subtypes of AML, including acute promyelocytic leukemia (APL). The most common cause of APL is a translocation between chromosome 15 and 17, t(15;17), that leads to the generation of the PML/RAR*α* fusion gene. The resulting fusion protein arrests the maturation of myeloid cells at the promyelocytic stage and leads to the increased proliferation of promyelocytes [143]. The cell lines typically used to study APL are NB4 and HL-60. NB4 has the t(15;17) translocation, while HL-60 does not [144]. In addition to chemotherapy and stem cell transplantation, treatments for APL also include differentiation therapy using all-*trans*-retinoic acid (ATRA) which has led to long-term disease-free survival in 70–80% of cases of this AML subtype [145].

An early study performed by Fujimura et al. demonstrated that treatment with troglitazone inhibited HL-60 cell growth by a G1 cell cycle arrest and induced their differentiation to monocytes [146]. A similar, G1 arrest was observed in all other hematopoietic cell lines examined. Furthermore, differentiation into the monocytic lineage was observed not only in the myelogenous and promonocytic cell lines, but also in an erythroleukemia cell line [146]. Data shown by Yamakawa-Karakida et al. demonstrated that PPAR*γ* activation by both troglitazone and 15d-PGJ_2_ inhibits proliferation and induces apoptosis in promyelocytic leukemia cells under serum-free conditions [147]. The induction of apoptosis was caspase-3 dependent, as treatment with a caspase-3 inhibitor completely abolished cell death. Although there were no changes in antiapoptotic or proapoptotic proteins, the expression levels of the proto-oncogene product *c-myc* were drastically reduced after 24 hours of troglitazone treatment while DNA binding by Tcf-4, a transcription factor responsible for *c-myc* expression, was completely inhibited [147]. Troglitazone and 15d-PGJ_2_ were found by Liu et al. to significantly induce apoptosis in K562 and HL-60 cells by upregulating the levels of the proapoptotic protein Bax and downregulating antiapoptotic proteins such as survivin and Bcl-2 [148]. Furthermore, these PPAR*γ* ligands downregulated the expression of cyclooxygenase-2 (COX-2), antiapoptotic proteins Bcl-2, Bcl-xL, and Mcl-1, upregulated Bax and activated caspase 3 in human monocytic leukemia cells [149]. Recent observations reported by Han et al. revealed that 15d-PGJ_2_ was able to sensitize tumor necrosis factor (TNF)-related apoptosis-inducing ligand (TRAIL)-resistant leukemic HL-60 cells to TRAIL-induced apoptosis [150]. This effect of 15d-PGJ_2_ was PPAR*γ*-independent, as treatment with a PPAR*γ* antagonist did not rescue the cells from apoptosis. These results were consistent with studies performed in other cancer cells where 15d-PGJ_2_ enhanced TRAIL-induced apoptosis [151]. In a human eosinophilic leukemia cell line, EoL-1, treatment with troglitazone caused a G_0_/G_1_ cell cycle arrest that correlated with increased mRNA levels of the cyclin-dependent kinase (cdk) inhibitor, p21WAF1/CIP1. Troglitazone exerted a similar induction of p21 mRNA accompanied by inhibition of cell proliferation in U937 cells and in the KPB-M15 human myelomonoblastic cell line [152]. These findings suggest that this PPAR*γ* ligand inhibits myeloid leukemia cell proliferation at least in part by upregulating p21 [152]. Aside from its growth inhibitory and apoptosis-inducing properties, 15d-PGJ_2_ has also been shown to decrease the expression of metalloproteinases in AML, therefore, inhibiting leukemic cell adhesion and invasion of the extracellular matrix (ECM) [153].

A recent study investigated the antileukemia effects and the molecular mechanism of action of a novel PPAR*γ* ligand, DIM#34, in AML. DIM#34 can inhibit cell growth and induce apoptosis through PPAR*γ*-dependent and -independent mechanisms. Cell death was associated with defective extracellular signal-regulated kinase (ERK) activity, and inhibition of Bcl-2 phosphorylation [154].

Konopleva et al. demonstrated growth inhibitory, differentiative, and apoptotic effects of PPAR*γ* ligands in cells from a variety of leukemias, including AML [155]. Addition of RXR or RAR ligands (i.e., LG100268 and ATRA, resp.) in combination with PPAR*γ* ligands enhanced the differentiative and growth-suppressive effects. Hirase et al. reported similar findings that the antiproliferative, proapoptotic, and/or differentiating effects of TZDs on HL-60 cells were further enhanced by the addition of the RXR-selective ligand, LG100268 [156]. PPAR*γ* ligands have also been shown to inhibit the clonal proliferation of U937 myelomonocytic leukemia cells by a G1 cell cycle arrest, and that treatment with both PPAR*γ* ligand (troglitazone) and LG100268 had synergistic inhibitory effects on clonal growth [157]. Finally, recent work by Yasugi et al. reported that both pioglitazone and 15d-PGJ_2_ inhibited cell proliferation in NB4 cells and that combined with ATRA, these PPAR*γ* ligands also induced myeloid differentiation and lipogenesis [158].

The PPAR*γ*-ligand CDDO and its C-28 methyl ester derivative (CDDO-Me) have also shown prodifferentiative properties in myeloid leukemia cells [159–161]. CDDO-Me induced granulo-monocytic differentiation in HL-60 cells and monocytic differentiation in primary AML cells. Interestingly, while colony formation of AML progenitors was significantly inhibited, normal CD34^+^ progenitor cells were less affected. The more potent effect of CDDO-Me on leukemic cells compared to normal progenitor cells suggests that CDDO-Me has potential as a new therapeutic agent for the treatment of hematological malignancies [159]. Another group found that low doses of CDDO promoted phagocytosis and granulocytic differentiation in HL-60 cells and primary blasts from AML patients through the regulation of CCAAT enhancer-binding protein (CEBPA) [162]. CEBPA is an important transcription factor for granulocytic differentiation. CDDO upregulated the transcriptionally active p42 CEBPA, while downregulating the inactive p30 CEBPA, thereby enhancing CEBPA-regulated gene transcription. These findings suggest the potential use of CDDO in the treatment of CEBPA-defective AML subtypes.

As proposed earlier, PPAR*γ* transactivation may be impaired in AML, and PPAR*γ* ligands may be able to sensitize AML cells to the prodifferentiation effects of ATRA [138, 139]. In light of this, a recent study revealed that CDDO enhanced ATRA-induced differentiation and apoptosis both in the ATRA-sensitive APL cell line, NB4, and an ATRA-resistant cell line, MR2 [163]. These effects were partially dependent on PPAR*γ*, as inhibition of PPAR*γ* either by a specific inhibitor (T007) or by siRNA diminished CDDO-induced APL differentiation [163].

CDDO induces apoptosis in human myeloid leukemia cells by promoting loss of mitochondrial membrane potential, leading to cytochrome c release and activation of caspases [155, 160, 162, 164]. However, Bcl-xL overexpression only partially inhibited cytochrome c release and caspase activation, indicating that CDDO can activate caspases 3 and 8 in a cytochrome c-independent manner [160]. Similar findings were shown by Konopleva et al. where CDDO activated both caspase-dependent and -independent cell death [164]. CDDO also promotes tumor necrosis factor (TNF)-induced apoptosis in human leukemia cells. CDDO exposure did not inhibit NF-*κ*B translocation into the nucleus, but rather inhibited a step after translocation, such as the NF-*κ*B-dependent resynthesis of the inhibitor of NF-*κ*B, I*κ*B*α* [165]. Similarly, Shishodia et al. demonstrated that CDDO-Me inhibited both constitutive and inducible NF-*κ*B activity in human leukemic cells. In contrast to the previous study [165], CDDO-Me-induced NF-*κ*B inhibition occurred through suppression of I*κ*B*α* kinase activation, I*κ*B*α* phosphorylation, I*κ*B*α* degradation, p65 nuclear translocation, and NF-*κ*B-mediated reporter gene transcription [166]. These results lead to a downregulation of NF-*κ*B target genes and enhanced apoptosis induced by TNF and other chemotherapeutic agents.

Another CDDO derivative, C-28 imidazole (CDDO-Im), appears to be more potent than CDDO in inhibiting the growth of human leukemia cells in vitro, as well as murine melanoma and leukemia cells in vivo [167]. The mechanism of CDDO and CDDO-Im-induced apoptosis has been attributed to a disruption of intracellular redox balance by increasing reactive oxygen species (ROS) and decreasing intracellular glutathione (GSH) concentrations [168].

Another subtype of AML is the acute myelomonocytic leukemia (AMML). A well established cell line derived from a child with AMML, THP-1, is often used to study this disease [169]. Several studies have shown that macrophages and myelomonocytic leukemias express PPAR*γ* and that PPAR*γ* agonists can induce differentiation of THP-1 cells into macrophages, as shown by the expression of CD36 scavenger receptors, as well as CD11b, CD14, and CD18 [97]. Another study showed that PPAR*γ*1 expression levels were upregulated by 9-*cis* retinoic acid (9-*cis* RA) in THP-1 cells coincident with suppression of cell growth [170]. Moreover, addition of a specific PPAR*γ* ligand enhanced 9-*cis* RA-induced growth inhibition [170]. A reduction in THP-1 cell migration also occurred in response to PPAR*γ* ligands and was due to downregulation of metalloproteinase-9 expression [171]. These findings suggest that PPAR*γ* ligands may be beneficial in preventing metastasis of monocytic leukemia cells. Indeed, PPAR*γ* ligands also have angiostatic properties because of their inhibitory effects on endothelial differentiation and on vascular endothelial growth factor (VEGF)-induced angiogenesis in vivo [172]. Recently, Ho et al. reported that the pigment epithelium derived factor (PEDF), a potent antiangiogenic factor, can induce THP-1 apoptosis and necrosis by inducing PPAR*γ* protein expression. In their study, PEDF-induced apoptosis was shown to be PPAR*γ*-induction-dependent. Treatment with PPAR*γ* antagonist and PPAR*γ* siRNA attenuated PEDF-induced apoptosis. Transient expression of PPAR*γ* using a PPAR*γ* expression plasmid reproduced the PEDF-effects. Importantly, the PPAR*γ* induced by PEDF was transcriptionally active. These results suggest a PPAR*γ*-dependent induction of apoptosis in THP-1 cells [173].

#### 5.1.2. Chronic myeloid leukemia (CML)

Chronic myelogenous leukemia (CML) is a myeloproliferative disorder that affects all hematopoietic cell types. It constitutes 15 to 20% of adult leukemias [174]. The American Cancer Society anticipated diagnosis of about 4570 new cases of CML in 2007 [174]. CML is characterized by a genetic abnormality known as Philadelphia (Ph) chromosome, resulting from a translocation between chromosomes 9 and 22, t(9;22)(q34;q11). This translocation generates a fusion protein called BCR-ABL which is a constitutively active tyrosine kinase responsible for uncontrolled cell proliferation and enhanced cell survival [175]. Treatments for this disease include splenic irradiation, stem cell transplantation, and interferon alpha (IFN*α*) administration with combination chemotherapy. A specific tyrosine kinase inhibitor, Imatinib, was introduced in the late 1990s and is a standard treatment for CML. However, clinical resistance to imatinib has been described in CML patients, where BCR-ABL gene mutations or amplifications have occurred [176, 177]. Therefore, development of new therapeutic strategies to overcome imatinib resistance is needed. Dual PPAR*α* and *γ* ligands have been tested, either alone or in combination with Imatinib, to overcome drug resistance. A characteristic cell line used to study CML is K562, which was established from a patient with CML in the acute phase [178]. Recently, a study was performed using a synthetic dual PPAR*α*/PPAR*γ* agonist, TZD18, in human CML myeloid blast crisis cell lines [63]. In this study, treatment with TZD18, both alone and in combination with Imatinib, inhibited CML proliferation and induced apoptosis. These effects were PPAR*α* and PPAR*γ*- independent, as neither PPAR*α* nor PPAR*γ* antagonists were able to rescue cell proliferation and survival. These results were reported previously by the same group in Ph-positive lymphocytic leukemia cell lines, where TZD18 promoted cell death and acted synergistically to enhance the effect of Imatinib [179]. Hirase et al. tested the effects of TZDs in K562 cells, which have an erythroid nature and the potential to differentiate into megakaryocytes [180]. TZD inhibited both cell proliferation and the erythroid phenotype of K562 cells. These results were accompanied by a reduction in erythroid lineage-transcription factor, GATA-1, levels [180]. Therefore, PPAR*γ* ligands may serve a therapeutic use for the treatment of other types of myeloproliferative disorders where there is an overproduction of erythrocytes, such as polycythemia vera (PV).

### 5.2. L ymphoid malignancies

#### 5.2.1. Acute lymphoblastic leukemia (ALL) and non-Hodgkin's lymphomas

Acute lymphoblastic leukemia (ALL) is a malignant disorder that arises from uncontrolled proliferation of lymphocytic progenitors. The disease is most commonly diagnosed in children, but can also occur in adults. About 80–90% of ALL patients can achieve complete remission with currently available therapy. Yet, many patients eventually relapse, and only 35% of individuals have a long-term leukemia-free survival (LFS) [181, 182]. Therefore, development of new treatment approaches to improve both the cure rate and the quality of life of patients with ALL is greatly needed. ALL involving hyperproliferation of B lymphocyte progenitors (B-ALL) is frequently associated with a translocation between the *c-myc* gene on chromosome 8q24 and any of the three immunoglobulin genes located on chromosomes 14q32, 2p11, or 22q11. This translocation results in *c-myc* overexpression and correlates with poor prognosis [183, 184]. The members of the Myc family, including *c-myc*, are involved in regulation of proliferation and development of normal and malignant cells [185].

An investigation by Zang et al. revealed that the PPAR*γ* ligands pioglitazone and 15d-PGJ_2_ suppressed cell growth in G1 phase and induced apoptosis in a dose-dependent manner in B-ALL cell lines. Apoptosis was found to be partly caspase-dependent, as treatment with a pan-caspase inhibitor partially reversed this effect [186]. Similar findings were shown in B-ALL with t(14;18), in which troglitazone not only induced G1 phase growth arrest and apoptosis, but also downregulated the expression of *c-myc* mRNA and protein [187].

Our group has demonstrated that: (1) both normal and malignant B lineage cells express PPAR*γ* mRNA and protein, and (2) exposure to certain small molecule PPAR*γ* ligands, including 15d-PGJ_2_, inhibits proliferation and induces apoptosis in these cells [85, 113]. Subsequently, we reported that PPAR*γ* ligand-induced apoptosis was mainly PPAR*γ*-independent, since it was not prevented either by a PPAR*γ* antagonist nor a dominant negative form of PPAR*γ* (PPAR*γ*-DN) [94]. We reported that the apoptotic mechanism regulated by 15d-PGJ_2_, but not by ciglitazone, was related to the production of ROS and the reduction in intracellular GSH [94]. CD40 signaling through CD40-ligand (CD40L) enhances B cell survival and prevents BCR-induced apoptosis by activating the transcription factor NF-*κ*B [188]. Therefore, we tested whether CD40 ligation could protect normal and malignant B cells from PPAR*γ* ligand-induced apoptosis. CD40L was able to partially rescue normal and malignant B cells from PPAR*γ* ligand-induced apoptosis by activating NF-*κ*B. Similarly, Piva et al. reported 15d-PGJ_2_-induced apoptosis in human Burkitt's lymphomas and multiple myeloma cell lines through inhibition of NF-*κ*B activity. These effects lead to the downregulation of NF-*κ*B-dependent antiapoptotic protein production and therefore decreased cell survival. The apoptotic effects could also be mimicked by NF-*κ*B p65 subunit knockdown by siRNA [189]. These results suggest a possible mechanism for the proapoptotic action of PPAR*γ* agonists.

We have also demonstrated that PPAR*γ* ligands can induce apoptosis in cells from human T cell leukemias (Jurkat), lymphomas (J-Jahn), and T-ALL cells (CCRF-CEM) by a PPAR*γ*-dependent mechanism [103]. Interestingly, normal T cells were not adversely affected by PPAR*γ* ligands, suggesting the use of PPAR*γ* agonists as selective therapeutic drugs for T-cell malignancies [103]. However, data from Yang et al. raised questions on the antiproliferative effects of PPAR*γ*-ligands in T-lymphoma cells [190]. They demonstrated that low concentrations of PPAR*γ*-ligands promoted T-lymphoma cell survival, while high concentrations promoted cell death. These results suggest that in T-lymphoma cells, PPAR*γ* ligands can have contradictory effects when used at different concentrations and require further examination.

Cutaneous T cell lymphoma (CTCL) is a group of T cell malignancies that accumulate in the skin. The most common CTCLs are (1) the Mycosis fungoides (MF), which develops as patches, plaques, or tumors containing apoptosis-resistant CD4^+^ CD45RO^+^ helper/memory T cells; and (2) the Sézary syndrome (SS), which is the leukemic form of CTCL that develops with erythroderma and the appearance of atypical T cells in the peripheral blood [191]. Current therapies for CTCL include the use of bexarotene, an RXR ligand, with good efficacy in the late stages of the disease [191]. Zhang et al. demonstrated the expression of PPAR*γ* in three CTCL lines (MJ, Hut78, and HH) and freshly isolated peripheral blood lymphocytes (PBL) from SS patients with circulating atypical T cells (CD4^+^CD26^−^) [192]. CDDO exposure caused a dose-dependent induction of apoptosis in MF/SS cell lines and SS patients' PBL [192]. These findings suggest that PPAR*γ* ligands may be beneficial for the treatment of CTCL and may have synergistic effects when used in combination with bexarotene.

Mantle cell lymphoma (MCL) is a rare type of non-Hodgkin's lymphoma (NHL), constituting about 6% of NHL [193, 194]. In 85% of MCL cases, a translocation between chromosome 11 and 14, t(11;14), is involved in the pathogenesis. This translocation leads to the overexpression of cyclin D1, a protein that increases cell survival and proliferation by positively regulating cell cycle entry into the S-phase [193]. Despite the success of current therapies, patients with mantle cell lymphoma have a shorter life span compared to patients with other B cell lymphomas [193]. Recently, a study demonstrated that treatment with pioglitazone and rosiglitazone, as well as with 15d-PGJ_2_ induced MCL cell apoptosis and downregulated cyclin D1 expression without altering cell cycle progression [195].

#### 5.2.2. Chronic lymphoblastic leukemia (CLL) and diffuse large B cell lymphoma (DLBCL)

CLL is a clinically heterogeneous disease originating from B lymphocytes that differ in activation, maturation state, or cellular subtype [196]. CLL is one of the most common forms of leukemia in adults [141]. In B-CLL, resistance to apoptosis has been associated with increased Bcl-2 expression, due to either promoter hypomethylation or to chromosomal deletion of the genes which encode two natural Bcl-2 antisense RNAs [197, 198].

To date, there are few studies that evaluate the use of PPAR*γ*-ligands against these malignancies. The effects of the triterpenoid CDDO were evaluated in refractory B-CLL cells. CDDO induced apoptosis in a dose-dependent manner in both previously untreated and chemoresistant CLL samples [199]. In this study, CDDO induced the activation of caspase-8, but not caspase-9, indicating the involvement of a mitochondrial-independent pathway [199]. CDDO also negatively affected the levels of an endogenous caspase-8 inhibitor, c-FLIP (caspase-8 homolog Fas-ligand interleukin-1-converting enzyme (FLICE)-inhibitory protein). However, downregulation of c-FLIP expression was not the sole pathway activated by CDDO, as c-FLIP antisense oligonucleotides did not induce CLL apoptosis [199]. Subsequently, Inoue et al. further investigated the mechanism of CDDO-induced apoptosis in primary B-CLL and Jurkat cell lines. In contrast to the studies discussed earlier [160, 164, 199], where CDDO activated both the intrinsic and extrinsic apoptotic pathways, Inoue et al. proposed that CDDO induces apoptosis exclusively through the intrinsic pathway [200]. In their study, CDDO exposure induced an initial caspase-independent mitochondrial depolarization, followed by caspase cleavage. Using caspase inhibitors, the authors were able to define caspase 9 as the primary activated caspase. Moreover, CDDO induced cell death in caspase-8 and FADD-deficient but not in Bcl-xL-overexpressing Jurkat T cells. In CLL, CDDO induced an initial release of proapoptotic intermediates, cytochrome c, and Smac/DIABLO from the mitochondria and led to apoptosis [200]. According to these results, CDDO mainly activates the intrinsic apoptotic pathway in both cell lines [200].

Diffuse large B-cell lymphomas (DLBCLs) are the most common lymphoid neoplasms, composing 30–40% of adult NHL [201]. The gene expression pattern (using DNA microarrays) of DLBCL was compared with that of normal B cells, including those from the germinal center (GC) and in vitro-activated peripheral blood B cells [202]. Based on the results, DLBCL were classified into two groups: those resembling B cells from the GC (GC-DLBCL) and those resembling in vitro-activated B cells (ABC-DLBCL). Patients with cancer of the GC-DLBCL-type have a more favorable prognosis than those with the ABC-DLBCL-type [202]. Although some DLBCL patients are cured with current therapies, most succumb to the disease. In addition, poor prognosis correlates with Bcl-2 overexpression, which may be responsible for the impaired apoptotic response of ABC-DLBCL to chemotherapy [203, 204].

Recently, a study by Ray et al. showed that CDDO induced growth inhibition and apoptosis in human DLBCL and that these effects were PPAR*γ*-independent [205]. Interestingly, CDDO induced NF-*κ*B activation and enhanced DLBCL apoptosis when combined with NF-*κ*B inhibitors. These findings suggest that NF-*κ*B may be activated as a survival pathway to antagonize the apoptotic effects of CDDO [205]. A recent study by Brookes et al. elucidated another mechanism for CDDO-induced cell death [206]. In this study, CDDO, CDDO-Im, and the dinitril derivative of CDDO, Di-CDDO induced both normal and malignant B cell apoptosis. The CDDO derivatives were more effective than CDDO itself. It was demonstrated that CDDO directly interacted with and modified several mitochondrial protein thiols, resulting in large molecular weight protein aggregates. These aggregates led to a loss in mitochondrial thiol status by constitutively opening cyclosporin A-insensitive permeability transition (PT) pores [206], thereby reducing mitochondrial transmembrane potential and resulting in cell death. These findings suggest a novel mechanism for triterpenoid-induced cell death and predict the development of new therapeutic drugs that can elicit unregulated PT pore formation in cancer cells.

### 5.3. Multiple myeloma

Multiple myeloma (MM) is a neoplastic disorder characterized by clonal proliferation of differentiated plasma cells in the bone marrow, accompanied by accumulation of monoclonal paraprotein levels in serum and urine. Common clinical symptoms include bone lesions, anemia, immunodeficiency, and renal failure [207]. MM constitutes ∼10% of hematological cancers and ranks as the second most frequent hematological malignancy in the United States after NHL [208, 209]. Current therapies for the disease include chemotherapy with or without stem cell transplantation, glucocorticosteroids, thalidomide, and the proteasome inhibitor Bortezomib (Velcade) and combinations of these agents. However, most of these treatments are not curative, and newer approaches are needed [209]. The therapeutic potential of PPAR*γ* ligands has also been evaluated in MM [13, 210, 211]. PPAR*γ* agonists have been demonstrated to have inhibitory effects in Waldenstrom's macroglobulinemia (WM), a rare plasma cell malignancy [212]. In addition, our laboratory demonstrated that human multiple myeloma cells modestly express PPAR*γ*. Treatment with PPAR*γ* ligands induced MM apoptosis via caspase activation and mitochondrial depolarization. These proapoptotic effects were not reversed by the addition of the MM growth factor IL-6. Moreover, we showed that these cells express RXR and that addition of an RXR ligand (9-*cis*-RA) enhanced PPAR*γ*-ligand-induced apoptosis [210]. Farrar's group found that PPAR*γ* ligands 15d-PGJ_2_ and troglitazone completely abolished IL-6-dependent MM cell proliferation and induced apoptosis. PPAR*γ* agonists inhibited MM cell survival by specifically blocking the IL-6-dependent transactivation of STAT3 (signal transducer and activator of transcription)-activated genes, including *c-myc* and *mcl-1* [13]. Recently, the same group has revealed that PPAR*γ* ligands inhibit (1) MM cell adhesion to bone marrow stromal cells (BMSC), (2) MM cell expression levels of adhesion molecules, and (3) BMSC secretion of IL-6, which is triggered by MM cell adhesion. The inhibitory effects of PPAR*γ* ligands correlated with PPAR*γ*-dependent transrepression of the transcription factors 5′-CCAAT/enhancer-binding protein *β* (C/EBP-*β*) and NF-*κ*B [213]. The PPAR*γ* ligands CDDO and CDDO-Im have also been tested in MM cells, both alone and in combination with the proteasome inhibitor PS-341 (Bortezomib) [214–216]. The mechanisms of CDDO-induced apoptosis include loss of mitochondrial membrane potential, which increases release of ROS and depletes glutathione, as well as activation of caspases and reduction of c-FLIP protein levels [214]. These results correlated with the studies described earlier, using CDDO in CLL [199]. Combination treatments of CDDO-Im with Bortezomib had synergistic apoptotic effects in MM cells [215], abolished NF-*κ*B and Bcl-2-mediated cytoprotective effects and overcame drug resistance to Bortezomib [215]. Overall, these findings suggest the use of CDDO-Im, either alone or in combination with bortezomib, to treat drug-resistant MM and improve patient prognosis.

## 6. CONCLUSIONS AND FUTURE DIRECTIONS

**Figure 3:**
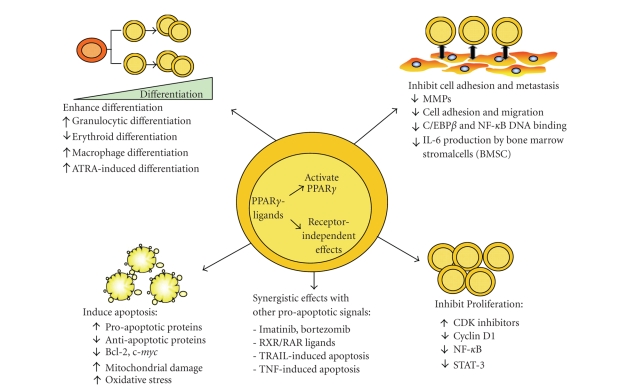
* Mechanisms of action of PPAR*γ* ligands in hematological malignancies.* PPAR*γ* ligands can bind to and activate PPAR*γ* to regulate gene transcription or they can exert PPAR*γ*-independent mechanisms. PPAR*γ* ligands have antiproliferative, prodifferentiation, antimetastatic, and proapoptotic effects on several hematological malignancies making them promising candidates for use in therapeutic regimens.

In summary, although the exact role of PPAR*γ* in controlling malignant cell growth and apoptosis remains unclear, PPAR*γ* has been commonly implicated as a tumor suppressor in hematological cancers (see Figure 3 for overview). Evidently, a better understanding of the mechanism of action of PPAR*γ* is needed. It is important that studies be performed to carefully analyze PPAR*γ* levels, as well as the activation status of PPAR*γ* in hematological cancers. In addition, since many of the existing studies have demonstrated that the proapoptotic and antiproliferative effects of PPAR*γ* ligands are independent of the receptor; additional studies are required to elucidate PPAR*γ*-dependent from independent events by using tissue specific knockouts, siRNA approaches, and overexpression studies. Understanding the mechanisms of action of these agents has become a priority to develop drugs that have beneficial effects on tumor suppression without having major side effects. Certain advances may be possible through the discovery of SPPAR*γ*Ms that can activate only a subset of desired genes. This will require the identification of PPAR*γ* target genes that mediate the antitumorigenic effects in hematological malignancies.

The fact that PPAR*γ* can be modified by phosphorylation through MAP kinases and that this modification decreases PPAR*γ* transcriptional activity, and the fact that PPAR*γ* activation itself increases PPAR*γ* degradation by the proteasome may be exploited for therapeutic benefit. PPAR*γ* ligands in combination with inhibitors of MAP kinases and/or proteasome inhibitors (e.g., Bortezomib) may be useful in the treatment of malignancy. Therefore, studies should be performed to assess the effectiveness of these combination therapies as well as those combining PPAR*γ* ligands with drugs such as Imatinib or RXR/RAR ligands. Our current knowledge of the anticancer potential of PPAR*γ* ligands predicts that such therapies may prove to be of great benefit for future treatments of hematological cancers.
